# Improved survival in both men and women with diabetes between 1980 and 2004 – a cohort study in Sweden

**DOI:** 10.1186/1475-2840-7-32

**Published:** 2008-10-20

**Authors:** Mats Eliasson, Mats Talbäck, Måns Rosén

**Affiliations:** 1 Department of Public Health and Clinical Medicine, Umeå University, Umeå, Sweden; 2 Centre for Epidemiology, National Board of Health and Welfare, Stockholm, Sweden; 3 Swedish Council on Technology Assessment in Health Care, Stockholm, Sweden; 4 Department of Internal Medicine, Sunderby Hospital, Luleå, Sweden

## Abstract

**Background:**

In Sweden, diabetes prevalence is increasing in spite of unchanged incidence, indicating improved survival. In recent US studies mortality in diabetic subjects has decreased over three decades, but only in men. Our aim was to study mortality over time in diabetic subjects.

**Methods:**

The annual Swedish Living Conditions Survey from 1980 to 2004 has been record-linked to the Cause of Death Register in order to study trends in mortality risk for those reporting diabetes as a chronic illness. Survival and the relative mortality risk within 5 years of follow-up have been calculated for a random sample of men and women aged 40–84 years with (n = 3,589) and without diabetes (n = 85,685) for the period 1980 to 2004. Poisson regression models were used.

**Results:**

The age-adjusted mortality risk relative to non-diabetics within 5 years of follow-up for men was doubled during all periods. The relative risk for women was initially about 2.5, with a substantial drop in mortality in 1995–1999 to 1.45 although it increased to 1.90 in the last period. Using models that took into consideration the presence of heart disease, hypertension, daily smoking, and socio-economic status at the initial interview did not change the relative mortality risk. The age-adjusted 10-year observed survival rate for men with diabetes increased from 41.4% 1980–1984 to 51.5% in 1995–1999. The observed survival for women increased from 43.7% to 61.0%.

**Conclusion:**

Survival rates have improved in subjects with diabetes since the early 1980s, more so in women than in men, thereby decreasing the gap to non-diabetic women.

## Background

Patients with diabetes have a markedly increased mortality which arises mainly from cardiovascular disease and end-stage renal disease [1-3]. The classical risk factors for cardiovascular disease, smoking, hypertension, and hypercholesterolemia, contribute strongly to the mortality [4]. Treatment methods for diabetes focus on improved glucose control and cardiovascular prevention. These methods have undergone several changes during the last decades, and international guidelines have been established.

There is a consensus among researchers that diabetes prevalence is increasing. The debate is rather whether this is due to an increasing age-specific prevalence due to an increase in incidence or to a longer longevity among diabetics [5] or whether a higher total prevalence can solely be ascribed to an aging population. Recent Swedish population studies do not support a higher incidence during the last 30 years [6,7] which corroborates pharmaco-epidemiological data from the Danish island of Fyn [8]. In Sweden, a higher prevalence over time seems restricted to the elderly [9]. Trends in all causes of death in Sweden where diabetes is either reported as an underlying or contributory cause of death show a clear sex difference, i.e. a small increase in diabetes mortality for men, but a steady decrease in mortality for women between 1970 and 2004 [9].

Conflicting results have been published concerning diabetes survival. Studies from the US and England have shown a clear improvement in diabetes survival up to 1999 [10,11]. A recent report from the National Health and Nutrition Examination Survey (NHANES) in the US showed that among diabetic men all-cause mortality decreased by more than forty percent between 1971 and 2000, accompanying the decrease in the non-diabetic population [12]. However, among women with diabetes, no decline in mortality was found, and that lead to a doubling of the mortality rate difference between diabetic and non-diabetic women. A recent report from the population based HUNT study in Norway described similar improvements in mortality in coronary heart disease among subjects with or without diabetes between the mid 1980's and the mid 1990's with a persistent doubled mortality risk in diabetic subjects [13].

The aim of this study was to report trends in diabetes survival in Sweden from 1980 to 2004 and to analyse whether these trends were different for the sexes and whether they were influenced by changes in risk factor patterns or by socio-economic status.

## Methods

Statistics Sweden has conducted annual surveys since 1975 of the living conditions of a random sample of about 7,000 people aged 16–84 years of age [14]. These surveys collect individual data on health, lifestyle, social and economic conditions, etc. It is possible to extract information from these surveys for those reporting diabetes as a chronic illness or if they regularly take medicines for diabetes. We linked records from the Living Conditions Surveys from 1980 to 2004 to corresponding records in the national Cause of Death Register from 1980 to 2004. This allowed us to compare survival among those reporting diabetes with survival among the rest of the survey sample (the general population) adjusting for heart disease, hypertension, smoking, and socio-economic status at the time of the interview (baseline). We restricted the analysis to the age group 40–84 years at the initial interview in order to obtain a more homogenous group of diabetes patients, mainly patients with Type 2 diabetes. The results presented here are based on 89,274 interviews: Table 1 presents baseline characteristics concerning the subjects. The average age of those reporting diabetes in the surveys was 65 years for men and 69 years for women and did not change over time.

**Table 1:** Base-line characteristics for subjects reporting diabetes as a chronic illness in the Swedish Living Conditions Survey 1980–2004.

		1980–1984		1985–1989		1990–1994		1995–1999		2000–2004	
		No	%	No	%	No	No	%	%	No	%
Diabetes among population											
Age 40–64	M	146	2.4	135	2.4	147	2.6	152	2.7	221	3.9
	F	91	1.5	98	1.8	86	1.5	106	1.8	140	2.3
Age 65–74	M	116	5.8	120	6.3	96	5.9	106	7.1	142	9.0
	F	130	5.9	126	6.0	101	5.7	130	7.8	129	7.3
Age 75–84	M	108	7.1	74	6.9	65	6.8	80	8.2	111	10.3
	F	166	8.3	117	7.9	122	9.8	96	7.8	132	8.9
CHD or hypertension^1 ^among diabetics	M	151	40.8	117	35.6	94	30.5	138	40.8	227	47.9
	F	214	55.3	150	44.0	132	42.7	145	43.7	219	54.6
CHD or hypertension^1 ^among non-diabetics	M	1 684	18.4	1 419	17.1	1 288	16.1	1 317	16.9	1 610	20.4
	F	2 274	22.9	1 632	18.6	1 405	16.5	1 445	17.2	1 738	19.6
Daily smokers among diabetics	M	101	27.3	71	21.6	69	22.4	70	20.7	73	15.4
	F	42	10.9	38	11.1	29	9.4	43	13.0	54	13.5
Daily smokers among non-diabetics	M	3 057	33.4	2 464	29.8	2 150	26.8	1 686	21.6	1 474	18.6
	F	1 989	20.0	1 929	21.9	2 026	23.8	1 863	22.1	1 768	19.9

Number of observations (No.) and percentages (%).

1. ICD-9: 401 – 429 Hypertension and coronary heart disease.

We used Poisson regression models with mortality as the dependent variable to analyse the relative mortality risk within 5 years of follow-up among diabetics and non-diabetics, for 5-year time periods from 1980–1984 to 2000–2004. The data for men and for women were analysed separately, and we included adjustments for age, reported heart disease and hypertension (ICD-9: 401–429), daily smoking, and socio-economic status level at the time of the interviews.

## Results

Multivariate analyses showed that the diabetics had a three- to four-fold higher mortality risk than non-diabetics (Table 2). Adjusting the results for age of the subjects reduced the relative risk, while adjusting for the other factors (heart disease, hypertension, smoking, and socio-economic status) did not change the relative risk. There was no substantial change over time in relative risk for men with diabetes as compared with men without diabetes. The diabetics' risk remained slightly above two throughout the period except for a small increase during the time period 1995–99.

**Table 2:** Relative mortality risk and 95% confidence intervals within 5 years of follow-up between subjects reporting diabetes as a chronic illness in the Swedish Living Conditions Survey 1980–2004 and those not reporting diabetes.

Sex/Period	Model 1	Model 2	Model 3	Model 4
Males				
1980–1984	3.28 (2.72 – 3.96)	2.04 (1.69 – 2.46)	2.08 (1.73 – 2.52)	1.93 (1.60 – 2.33)
1985–1989	3.46 (2.81 – 4.26)	2.13 (1.73 – 2.63)	2.17 (1.76 – 2.68)	2.07 (1.68 – 2.55)
1990–1994	3.52 (2.80 – 4.44)	2.28 (1.81 – 2.88)	2.29 (1.82 – 2.89)	2.22 (1.76 – 2.79)
1995–1999	4.11 (3.26 – 5.17)	2.51 (2.00 – 3.17)	2.54 (2.02 – 3.20)	2.42 (1.93 – 3.05)
2000–2004	3.35 (2.60 – 4.30)	2.09 (1.63 – 2.69)	2.12 (1.65 – 2.73)	1.97 (1.53 – 2.53)

Females				
1980–1984	4.52 (3.72 – 5.49)	2.47 (2.03 – 3.00)	2.51 (2.07 – 3.06)	2.39 (1.97 – 2.91)
1985–1989	5.05 (4.07 – 6.25)	2.77 (2.24 – 3.43)	2.80 (2.26 – 3.47)	2.69 (2.17 – 3.33)
1990–1994	5.35 (4.20 – 6.81)	2.43 (1.91 – 3.10)	2.49 (1.96 – 3.18)	2.44 (1.91 – 3.11)
1995–1999	2.77 (2.04 – 3.76)	1.45 (1.07 – 1.98)	1.47 (1.08 – 2.00)	1.41 (1.04 – 1.92)
2000–2004	3.52 (2.61 – 4.75)	1.90 (1.40 – 2.56)	1.92 (1.42 – 2.59)	1.80 (1.33 – 2.43)

Model 1: crude model				
Model 2: controlling for age				
Model 3: controlling for age, daily smoking, and socioeconomic status				
Model 4: controlling for age, daily smoking, socioeconomic status, CHD and hypertension^1^				

Non-diabetics as reference.

1. ICD-9: 401–429 Hypertension and coronary heart disease.

The relative risk for women, adjusted for age, was 2.47 in 1980–1984, 2.77 in 1985–1989, and 2.43 in 1990–1994, followed by a substantial drop to 1.45 in 1995–1999 (Table 2). Mortality risk increased to 1.90 in the last cohort. After adjusting for the full model (model 4), the 1995–1999 cohort only barely differed from the non-diabetic population (RR 1.41; CI 1.04–1.92). On the other hand, this period had a significantly lower relative mortality risk than the two earlier periods.

**Figure 1:**
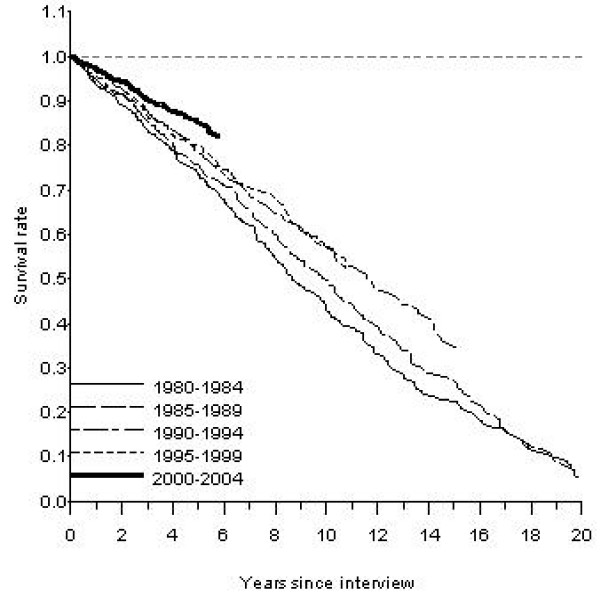
**Age-adjusted observed survival rates for men reporting diabetes as a chronic illness in the Swedish Living Conditions Survey 1980–2004.** Adjusted to the age of the diabetic subjects.

**Figure 2:**
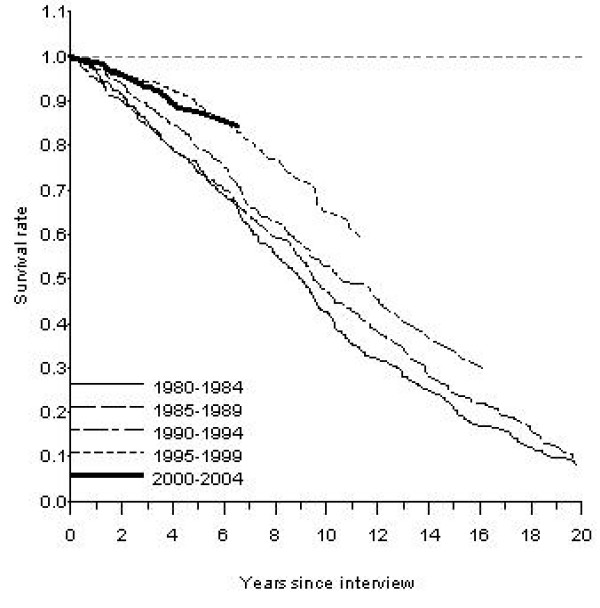
**Age-adjusted observed survival rates for women reporting diabetes as a chronic illness in the Swedish Living Conditions Survey 1980–2004.** Adjusted to the age of the diabetic subjects.

Figures 1 and 2 present long-term survival rates for men and women in Sweden for those reporting diabetes as a chronic illness in the surveys. For each consecutive cohort some further improvement in survival can be discerned. For men the most obvious improvement was for the latest cohort and for women the latest two cohorts.

The long-range observed survival rates for diabetics improved substantially (Table 3). Five year survival increased from 68.4% to 80.4% in men between 1980–1984 and 2000–2004 and for women from 71.9% to 85.2%. For the cohort reporting diabetes in 1980–1984, the age-adjusted 10-year observed survival rate was 41.4% in men and 43.7% in women Men and women reporting diabetes fifteen years later, in the survey covering 1995–1999, had a 10-year survival rate of 51.5% and 61.0%, respectively.

**Table 3:** Age-adjusted observed survival rates (%) for males and females reporting diabetes as a chronic illness in the Swedish Living Conditions Survey 1980–2004.

Sex/Follow-up	1980–1984	1985–1989	1990–1994	1995–1999	2000–2004
Males					
5 years	68.4	69.2	71.3	73.9	80.4
10 years	41.4	46.6	51.0	51.5	
15 years	26.0	30.3	34.5		
20 years	14.9	15.7			
Females					
5 years	71.9	72.3	75.5	86.4	85.2
10 years	43.7	46.9	50.3	61.0	
15 years	26.6	29.8	35.2		
20 years	15.8	19.1			

Adjusted to the age of the survey population.

Fifteen years survival rates have improved from about 26% to about 35% among both men and women. The age-adjusted survival rate after 20 years of follow-up of the 1985–1989 cohort was 15.7% in men and 19.1% in women

## Discussion

There are few studies of time trends in long-term survival among a population-based non-selected sample of subjects with diabetes. In general, and as expected, diabetic subjects have a higher mortality risk than the general population for the whole period 1980 to 2004. On the other hand, survival rates have improved among diabetics in parallel with the non-diabetic population. Women with diabetes may even have improved their survival to a greater degree than non-diabetic women and thereby possibly decreased the gap.

We found that the relative mortality risk was doubled, and that is consistent with many other published studies. It is, however, somewhat surprising that the risk persisted and was unchanged even after adjusting for the presence of heart disease and hypertension, smoking habits, and socio-economic status. The unchanged risk after adjusting for heart disease and hypertension may be a result of diabetic subjects being identified and more intensively treated for heart disease and hypertension to a higher degree than non-diabetics. The non-diabetics may very well be unaware of their cardiovascular risk. However, this cannot explain why well-known risk factors such as smoking and socio-economic status did not affect the relative risk, but the impact of diabetes as an independent risk factor may well be so strong as to attenuate the effect of other risk factors. Interestingly, the HUNT study showed no change in cardiovascular risk after adjustment for multiple risk factors [13]. The small changes in relative risks when adjusting for socio-economic status may be a result of the high level of equity of access to medical care in Sweden.

The observed 10-year survival rate for men with diabetes has improved modestly with 10 percent units from the cohort diagnosed in 1980–1984 as compared with the cohorts followed since the mid and late 1990s. Still, impressive further improvement now is discerned in the 5-year mortality in the 2000–2004 cohort. However, there was no improvement in relative mortality in diabetic men as compared with those without diabetes between 1980 and 2004. These finding fit in well with the recent report from NHANES and the HUNT study [12,13]

On the other hand, one encouraging result was the substantial increase in 5- and 10-year survival rate for women with diabetes, 13 and 17 percent units, respectively, which primarily occurred in 1990s and was also discernible, although not as impressive, in the latest cohort. The relative mortality risk for diabetic women during the time period 1995–1999 was only 41 percent higher than in non-diabetic women. The higher risk noted in the period thereafter (although far lower than at baseline) must raise the possibility of a spurious finding, but in the light of the survival curves a more beneficial development in women than in men is likely. Our findings, thus, are at odds with NHANES' data where no decrease at all in mortality rates of diabetic women was found between 1971 and 2000 [12]. On the other hand, the Norwegian HUNT study noted similar descreases in cardiovascular mortality in women with or without diabetes on long term follow up of two cohorts initiated 1985 and 1996 [13].

A Canadian study showed a 25% reduction in diabetics' mortality between 1995 and 2005 [15]. A Finnish study showed that diabetes survival during the 1990s depended on socio-economic differences, while survival was independent of socio-economic status in the 1980s [16]. In general, diabetic subjects in lower socio-economic groups have poorer metabolic control, report more complications, and have lower survival rates than diabetics in higher socio-economic groups [16-18].

Improved survival of Swedish diabetics is most probably the effects of significant changes in treatment since the early 1980s with focus on cardiovascular prevention. Most prominent is the aggressive treatment of hypertension, especially with ACE inhibitors [19,20] and more recently, with lipid-lowering statins [21,22]. The introduction of thrombolysis following acute myocardial infarction also gave impressive reductions in the mortality of patients with diabetes. Diabetic patients after their first acute myocardial infarction have as miserable a prognosis as that of non-diabetic patients after a recurrent infarction [23]. That led to the practice of physicians adopting a strategy of secondary prevention, treating supposed IHD in diabetic patients before overt symptoms appeared. Efforts to achieve improvement in glucose balance are difficult to assess and their effect on survival is less proven as highlighted by the recent results from the ADVANCE and ACCORD studies [24,25]

Thus, evidence for the success of cardiovascular prevention in diabetic patients accumulated over a period of time, and was probably not extensively implemented in primary care (where almost all Type 2 diabetic patients are treated in Sweden) until the latter half of the 1990s. The full impact of these measures is therefore probably not yet appreciated.

A crucial question is whether diabetics have the same decrease in the incidence and mortality in cardiovascular disease as the general population and whether similar gender differences in trends are noted. Such findings would strongly support our data. The improved survival for women with diabetes during the late 1990s is supported by mortality data from an independent source, the Cause of Death Register [9]. In a report from the Northern Sweden MONICA study, subjects with diabetes did not reduce the incidence of myocardial infarction as compared to non-diabetic subjects between 1989 and 2000, although the improvement in case-fatality did not differ significantly between groups [26]. Women with diabetes had a tendency towards decreasing incidence and mortality in first-infarction, although non-significantly. A similar analysis of stroke incidence and survival from the same MONICA centre showed that women with diabetes decreased their risk of stroke, relative to non-diabetics, but diabetic men did not [27]. Mortality rates improved to a similar extent in both men and women with diabetes and in parallel to non-diabetics. These data support both our findings of decreasing mortality among all diabetics and that of different time trends for men and women with diabetes.

Then why have male diabetics not experienced the same decrease in mortality? One hypothesis is that women comply to a larger extent than men with a doctor's advice. However, one American study did not find any gender differences in care adherence between men patients and women patients with diabetes [28]. Further, a Swedish study showed that there is no gender difference in the level of glycemic control, although diabetic women visit outpatient clinics more frequently than diabetic men. That would give the women a greater opportunity to vigorously treat high blood pressure and cholesterol levels [29]. The more frequent contact of women with health care in general may result in diabetes being diagnosed at an earlier and milder stage in women than in men, giving higher survival rates. Differences in smoking rates may explain some of the gender differences; up to recent years we found that female diabetics smoke much less than male diabetics.

Some factors must be considered when considering the conclusions that we have drawn. One limitation lies in the identification of subjects. The study group consists of those who either report diabetes as a chronic disease or who report they are treated with diabetes drugs. This means that the control group contains some undiagnosed cases of diabetes as no sampling for plasma glucose was done. Another factor concerns the type of diabetes from which a subject suffers. We have not been able to differentiate between Type 1 and Type 2 diabetes, nor do we know the ages at diagnosis. The duration of the disease has a clear effect on survival. On the other hand, the statistical surveys are based on random samples of the population, and there are no indications that the median age at diagnosis has changed significantly over time. Further, we have adjusted the results for the age of the subjects. A minor drawback of our design is that subjects with diabetes below 40 years of age are not included but our data are valid for the vast majority of Swedish diabetic subjects.

This study should be followed up by at least two measures. Firstly, more studies are needed in order to confirm or refute the tendency to increased survival of women with diabetes. Secondly, the somewhat discouraging results for men show that clinicians and general practitioners must intensify their efforts to improve the evidence-based primary and secondary preventive measures for patients with diabetes. They must also intensify their efforts to increase the adherence of patients to treatment regimens.

## Competing interests

The authors declare that they have no competing interests.

## Authors' contributions

ME contributed to the analysis and the interpretation of the data, the written material, and finalised the draft. MT was responsible for data management and the statistical analyses and contributed with written material and comments to the final draft. MR had the idea for the study, wrote the first draft and contributed to the analysis and interpretation of the data and comments to the final drafts
